# The TSANZ Practical Guide for Clinicians in the Management of Screen‐ and Incidentally‐Detected Nodules

**DOI:** 10.1111/resp.70065

**Published:** 2025-05-29

**Authors:** Fraser Brims, Annette McWilliams, Jonathan Williamson, Miranda Siemienowicz, Tracy L. Leong, the Thoracic Society of Australia & New Zealand Lung Cancer Working Party, Asha Bonney, Vanessa Brunelli, Archit Chawla, Paul Dawkins, Teresa Fae, Kwun Fong, Catherine Jones, Steven James Lindstrom, Henry Marshall, Mohan Nagarajah, Phan Nguyen, Gerard Olive, Diane Pascoe, Calvin Sidhu, Daniel Steinfort, John Taverner, Dhaval Thakkar, Patricia Wenzlick

**Affiliations:** ^1^ Curtin Medical School Curtin University Perth Western Australia Australia; ^2^ Department of Respiratory Medicine Sir Charles Gairdner Hospital Perth Western Australia Australia; ^3^ Institute for Respiratory Health Perth Western Australia Australia; ^4^ Department of Respiratory Medicine Fiona Stanley Hospital Perth Western Australia Australia; ^5^ School of Medicine University of Western Australia Crawley Western Australia Australia; ^6^ Faculty of Medicine, Health and Human Sciences Macquarie University Sydney New South Wales Australia; ^7^ Department of Respiratory Medicine Liverpool Hospital Sydney Australia; ^8^ Northern Imaging Victoria Northern Health Epping Victoria Australia; ^9^ Department of Radiology Alfred Health Melbourne Victoria Australia; ^10^ Central Clinical School Monash University Melbourne Victoria Australia; ^11^ Department of Respiratory and Sleep Medicine Austin Health Melbourne Victoria Australia; ^12^ University of Melbourne Melbourne Victoria Australia; ^13^ Olivia Newton‐John Cancer Research Institute Melbourne Victoria Australia

**Keywords:** incidental pulmonary nodule, indeterminate pulmonary nodule, lung cancer, lung cancer screening

## Abstract

The increasing adoption of lung cancer screening programs and advancements in imaging technologies has significantly increased the detection of pulmonary nodules, both incidentally and through screening. This document provides a comprehensive guide for clinicians to address the complexities of managing indeterminate pulmonary nodules (IPNs), emphasising person‐centred and multidisciplinary care. IPNs are categorised based on size and morphology, with specific guidelines for malignancy risk stratification, diagnostic evaluation, and follow‐up. Dedicated lung nodule evaluation teams (LNETs) and nodule multidisciplinary meetings (MDMs) play a critical role in ensuring guideline adherence, streamlining the diagnostic pathway, reducing unnecessary investigations, and improving outcomes. Structured IPN programs have demonstrated benefits in early lung cancer detection, improved detection of early‐stage lung cancer, and reduced delays to treatment initiation. Effective management strategies include use of standardised reporting templates, utilising validated risk models such as the PanCan malignancy risk model and agreed protocols for follow up of IPNs. This document highlights the importance of accessing prior imaging to assess for growth and accounting for technical differences between computed tomography (CT) scans. Any nodule considered to be growing requires discussion at a nodule MDM with decision to act for tissue biopsy as appropriate. A nodule MDM will assist in optimising the safest and most efficient biopsy techniques based on nodule characteristics and risk profile. By integrating multidisciplinary expertise and adhering to evidence‐based protocols, services can improve the timely diagnosis and management of IPNs, minimise over‐investigation, reduce chance of overdiagnosis and ultimately enhance patient outcomes and lung cancer survival.

## 1 Introduction

The adoption of lung cancer screening in many countries and the increasing numbers of incidental pulmonary nodules detected as part of imaging the thorax for other reasons has led to growing interest in dedicated nodule evaluation pathways and teams. With a rapidly changing evidence base for nodule management, there is an increasing need for adaptation of clinical services, with innovations such as virtual lung nodule clinics, establishing a specialist lung nodule evaluation team (LNET) and nodule multidisciplinary meetings (MDMs) within a respiratory service.

### 1.1 Nodule Terminology

**FIGURE 1:**
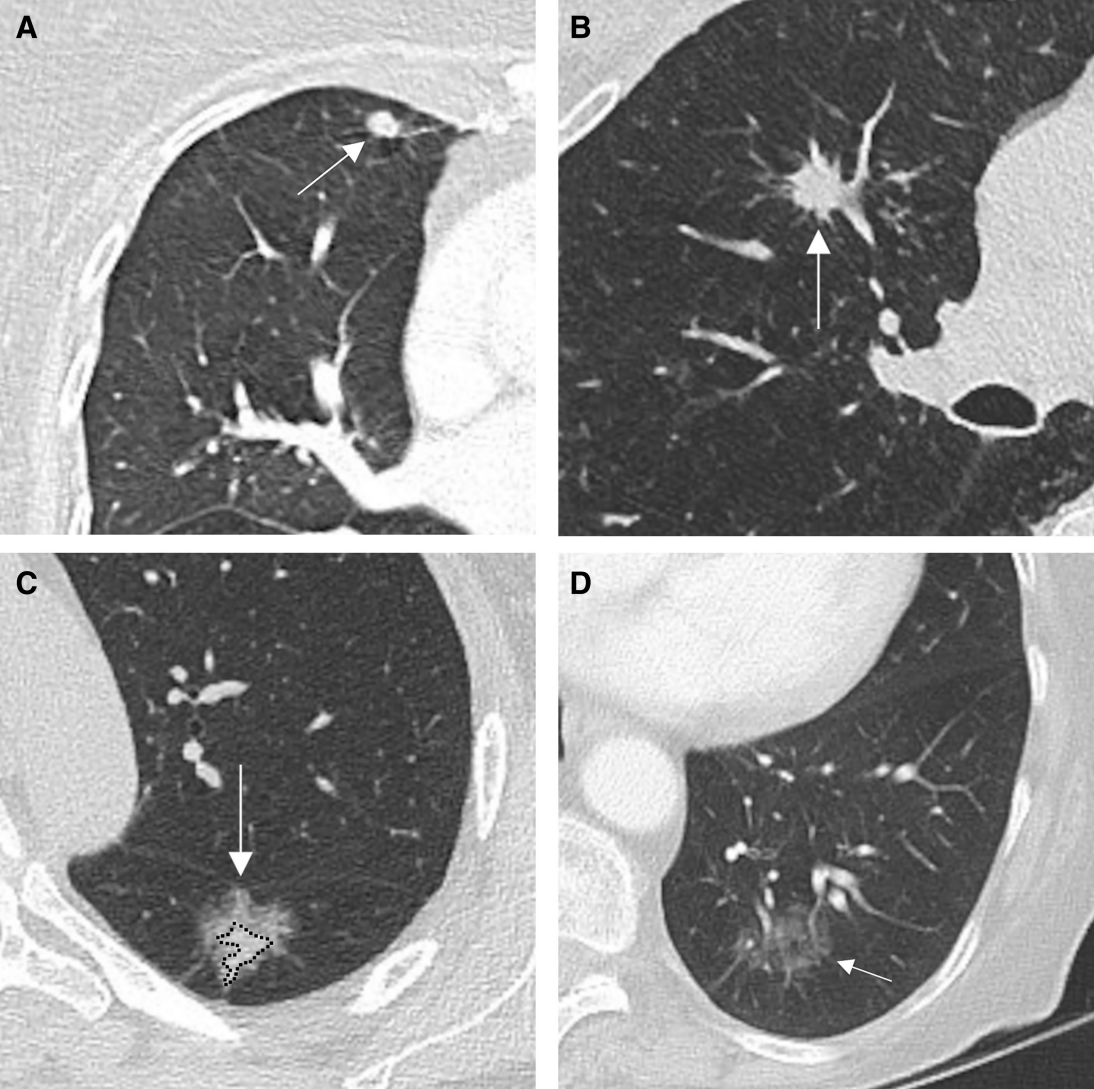
(A) Axial post‐contrast CT. Eight millimetre indeterminate pulmonary nodule in the right upper lobe (white arrow), solid with a smooth margin and subtle microlobulation. (B) Axial non‐contrast CT. Eleven millimetre solid nodule in the right upper lobe (white arrow). The spiculated margin increases the radiological likelihood of malignancy. (C) Axial non‐contrast CT. Twenty millimetre part solid nodule in the left lower lobe (white arrow). Both the ground glass and solid (dotted line) components have complex margins, limiting reproducibility of manual measurement and accuracy of automated volumetry. Direct visual comparison is critical to determine change over time. (D) Axial post‐contrast CT. Twenty‐five millimetre non‐solid nodule in the left lower lobe (white arrow). Blood vessels are visible through the lesion, hence non‐solid.

**FIGURE 2:**
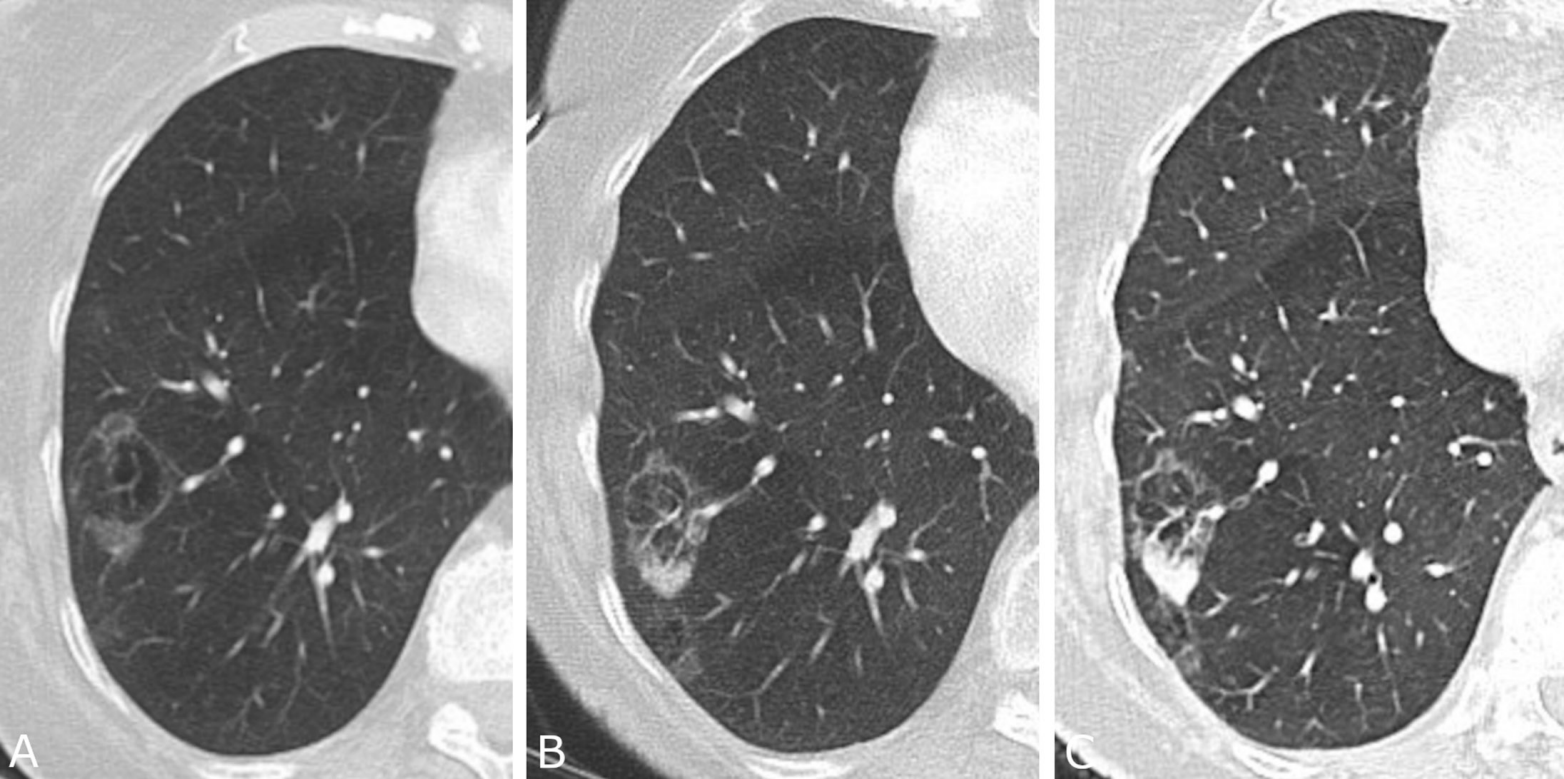
Progression of an atypical pulmonary cyst. Post‐contrast axial CT scans performed at baseline (A), 13 months (B) and 19 months (C) show progression of a peripheral opacity from ground glass to solid. There is no change in overall lesion size.

**FIGURE 3:**
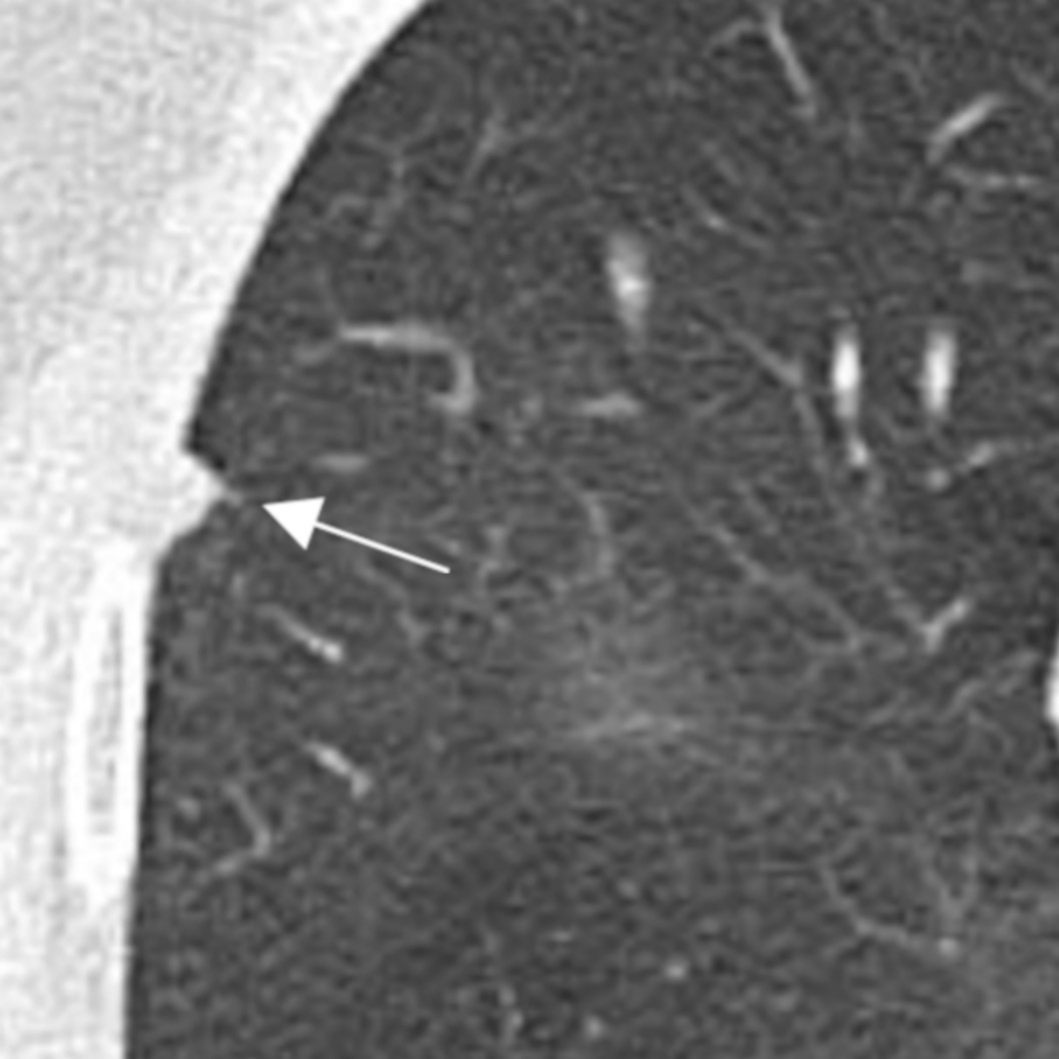
Axial non‐contrast CT. Eight millimetre juxtapleural nodule in the right middle lobe (white arrow). Triangular morphology, position relative to the pleura and size less than 10 mm indicate benignity. This reflects a normal intrapulmonary lymph node.

A lung nodule is defined as a discrete lung parenchymal lesion < 30 mm (a mass defined as ≥ 30 mm). A non‐solid (ground glass) nodule is a focal nodular area of increased lung attenuation on a computed tomography (CT) scan through which normal parenchymal structures (i.e., airways and vessels) can be visualised [1, 2, 3, 4]. These are pure ground glass, with no solid component. A part solid nodule is a discrete lung parenchymal nodule with both a ground glass and a solid component [1, 2, 3, 4]. See Figure 1 for examples of solid, part solid, and non‐solid nodules. Atypical pulmonary cysts have thickened walls > 2 mm, internal septations, or associated nodules or other opacities [1, 2, 3, 4], see Figure 2. Juxtapleural (perifissural) nodules meeting location and morphological criteria correspond to intrapulmonary lymph nodes and may be regarded as benign (Figure 3) [5]. These should be < 10 mm, solid, with smooth margins and oval, lentiform, or triangular in shape; interrogation in multiple planes is required. These nodules should contact the pleura (any location) or may be within 15 mm of the pleura when in the middle or lower lobes [6]. Further relevant terminology and other features of benign nodules are summarised in Boxes 1 and 2.

BOX 1 **Core staffing and terminology for nodule teams. EBUS, endobronchial ultrasound; IPN, indeterminate pulmonary nodule; MDM, multidisciplinary meeting.**

1

**Table:** 

Recommended staffing for lung nodule evaluation team (LNET)	
Core LNET staff	Care coordinator Respiratory physician with experience in interventional (EBUS) bronchoscopy Radiologist with experience thoracic diagnostic and interventional (biopsy) procedures Specialist nurse
Optional LNET staff	Thoracic surgery Radiation Oncology Trainees in respiratory, radiology
Important terminology for managing pulmonary nodules	
Indeterminate Pulmonary Nodule	A nodule where the probability of malignancy is uncertain but does not require immediate investigation
Screen detected nodule	A nodule detected as part of a formal lung cancer screening program
Incidental nodule	A nodule detected on a CT scan performed for a different indication
Lung Nodule Clinic	A dedicated clinic for IPN management. May be virtual, face to face or hybrid
Lung Nodule Evaluation Team	A specialist team for management of IPNs
Baseline scan	The first scan performed as part of a formal screening program
Index scan	The earliest CT scan demonstrating an incidentally detected nodule. Serves as the start of the surveillance period.
Fast Track Clinic	Rapid access evaluation clinic for suspected lung cancer
Nodule MDM	Dedicated multidisciplinary meeting for nodule management. May be virtual, face to face or hybrid
Thoracic cancer MDM	Thoracic cancer multidisciplinary meeting with full, wider staffing representation

BOX 2 Clinical information to support the evaluation of an indeterminate pulmonary nodule. *Note: Risk brackets and suggested management differ from the NLCSP.

1

**Table:** 

**Benign nodules**—nodules that *do not require referral* or specialist assessment include:	
Nodules with diffuse, central, laminated or popcorn patterns of calcification or macroscopic fat Juxtapleural (perifissural) nodules with characteristic morphology < 10 mm diameter Solid nodules stable for at least 2 years Non‐solid, part solid and atypical pulmonary cysts stable for at least 5 years	
Highly desirable information required for incidental nodule evaluation	
Patient characteristics	Patient history, symptoms and indication for CT (if CT performed for respiratory (infective) symptoms, consider short interval repeat CT depending on radiological likelihood of malignancy (e.g., 8–12 weeks)
	Detailed smoking history including tobacco, marijuana, electronic cigarettes and illicit drugs
	Family history of lung cancer
	Ethnicity
	Occupational exposures
	Known underlying lung disease, for example, COPD, Interstitial lung disease
	Personal history of cancers
	Medications, for example, anticoagulation, immunosuppressive drugs
**CT Scan characteristics**	Screen‐detected or incidental nodule
	Current and prior chest CT imaging: date and radiology provider
	Radiology report(s)
Recommended incidental nodule malignancy risk and follow up*	
PanCan malignancy risk of nodule	Recommended interval CT period
< 6%	12 month
6%–30%	3 month
> 30%	Immediate fast track clinic assessment

Outcomes from triage/initial review	
Document nodule type (solid, part solid, non‐solid, cystic) Document nodule risk (PanCan%/*Fleischner* (low/high risk)/benign) Follow up plan (discharge/surveillance period: 2 years, 5 years) Document next planned CT date and which provider Document planned clinic review—face to face/telephone/telehealth Decision to Act (high risk, definite growth/change)—refer to Nodule MDM Ensure clear communication with patient and referring practitioner

Incidental nodules are detected on CT conducted for a non‐nodule indication, such as CT pulmonary angiogram [7]. There should be no associated mediastinal lymphadenopathy or other features concerning for malignancy. In Australia, there has been a 200% increase in the number of CT chest scans performed between 2001 and 2019 [8] and incidental nodules are detected in 2.5%–56.3% of CT chests performed (within other populations) [7, 9, 10].

An indeterminate pulmonary nodule (IPN) is defined as a nodule where the ‘probability of malignancy is uncertain (suspicious, not very low or very high)’, [11] equating to a nodule that does not require immediate investigation, nor discharge. Whilst this recent definition is for screen detected nodules, it lends itself to incidentally detected nodules, in addition.

In Australia, the National Lung Cancer Screening Program (NLCSP) Nodule Management Protocol will utilise a risk‐based approach to IPN management and will only refer patients to respiratory specialists as part of a multidisciplinary team when a nodule is high (category 5) or very high (category 6) risk of malignancy [12]. These high and very high risk screen‐detected nodules may have a positive predictive value of malignancy of 48% [13], in a population already selected as high background risk. This contrasts with individuals with incidentally detected nodules that may have a variable background risk of malignancy; consequently, a variable malignancy rate of the nodules, with reports ranging from 5% to up to 25% malignancy rates [10, 14, 15, 16]. This risk is highly dependent on the demographics and risk profile of the population(s).

The optimal management of IPNs involves timely review, adherence to established guidelines, and a multidisciplinary approach to investigation and management. Dedicated nodule teams, nodule assessment clinics, and nodule MDMs will likely help facilitate optimal care.

## 2 Evidence Appraisal and Recommendations

The TSANZ Lung Cancer Working Party (LCWP), comprised of experts from Australia and New Zealand in respiratory, radiology and nursing, and including consumer representation, held a series of face‐to‐face and virtual meetings in 2024. The LCWP identified clinically relevant questions based on their own expertise and knowledge of the literature, existing guidelines and known knowledge gaps. With the aid of a medical librarian, literature searches were performed from January 1, 2000 to August 31, 2024 using combinations of words or terms that included the scope of the project.

The relevant literature was evaluated by the panel of experts with the aim of summarising into a narrative practical guide, with the proposed key messages based on scientific evidence and expert opinion. As this project was not a formal systematic review or meta‐analysis, graded recommendations are not provided. The final manuscript was derived from multiple meetings and reviews with consensus from the LCWP and approval from TSANZ.

### 2.1 Target Audience

This document is intended for respiratory physicians, radiologists, and those involved in the service delivery of fast‐track clinics, lung nodule clinics, and nodule MDMs. The subject matter is Australasian‐centric, but many of the core principles will be relevant for clinicians more widely.

## 3 The Role of Lung Nodule Programs and Clinics

The evaluation and investigation of an IPN should follow the guiding principles of person‐centred and multidisciplinary care, timely access to evidence‐based pathways, efficiency and accuracy, and coordination, communication and continuity of care [17]. Specialist nurses (clinical nurse consultants) provide a critical role in this field of practice [18].

Dedicated lung nodule programs and clinics for incidentally detected nodules may increase early detection of lung cancers outside of formal lung cancer screening programs. Such programs may have a different patient cohort to lung cancer screening participants, with up to 51% of those with incidentally detected lung cancer not meeting screening eligibility criteria [19, 20]. In a US‐based study, the value of an IPN program was exemplified by increasing stage 1 and 2 lung cancer diagnoses from 23% to 36% in its first year [9]. A similar program found that IPN program implementation led to a reduction in average days to treatment from 41 days to 28 days [16]. Further, IPN‐program diagnosed lung cancer patients had a better 5 year survival compared with the non‐program cases [21].

Unstructured IPN management can involve a number of challenges such as inconsistent follow‐up, unnecessary diagnostic procedures with associated risks and complications, and concerns suffered by patients [19]. Therefore, a structured IPN program can help to standardise care, improve patient outcomes, and optimise resource utilisation. The approach chosen must consider the potential benefits of timely intervention against the potential harms of diagnostic procedure complications, unnecessary procedures on benign disease, or delay in definitive treatment. Studies have shown nodule MDMs can effectively streamline follow‐up processes, reduce disparities in care, and can increase guideline‐concordant decisions [14, 22, 23].

Clinical guidelines are typically utilised by clinicians to aid in risk stratification and decision making regarding IPN management, such as the American College of Chest Physicians [24] and British Thoracic Society [25]. More recently, guidelines have been developed specifically for incidental nodules (Fleischner Society) [3] and screen‐detected nodules (Lung‐RADS) [4]. These provide recommendations for follow‐up and consideration of additional management [10, 20]. However, these guidelines have limitations as they cannot be applied to all clinical scenarios including patients with active infection, known history of malignancy, and age < 35 years. Furthermore, there may be variability in radiology reporting depending on reporting setting. Retrospective cohort studies estimate guideline concordant radiology report recommendations may vary significantly [26, 27, 28, 29], although in the setting of collaborative clinical pathways and formal IPN tracking systems, guideline concordant reporting improves to around 86.6% [30].

Cost considerations are an important aspect of establishing IPN programs. Adhering to guidelines to manage IPNs can reduce costs by helping promote less intensive nodule evaluations and reducing adverse events, all without compromising cancer diagnosis and staging [31]. This same study demonstrated that in the group who received non‐guideline concordant care, there was a higher cost (almost double) and greater radiation exposure to the patients [31]. Electronic tracking systems and standardised reporting have also been shown to reduce costs by facilitating appropriate follow‐up intervals and reducing redundant tests [32].

### 3.1 Core Clinical Information Needed for a Lung Nodule Clinic

It should be noted that NLCSP Category 5 and 6 screen‐detected IPNs will have a high risk of malignancy. For incidental nodules, the risk of malignancy is dependent on the clinical context of the chest CT, the individual patient risk factors, CT nodule characteristics, and other CT findings. Evaluation of all these factors is required to provide appropriate advice and recommendations regarding either ongoing CT surveillance or seeking a definitive diagnosis following a decision to act. Table 2 details the minimum desirable clinical information required to assist with the evaluation of an IPN at initial assessment or triage of referral.

### 3.2 Staffing: Lung Nodule Evaluation Team (LNET)

The role of a LNET should be to provide specialist expertise to reduce over/under‐investigation, reduce missed lung cancers, rationalise management options within local service constraints, educate, improve the quality of patient care, and improve guideline concordant care [23, 33].

Specialist expertise is required from initial triage of referral through to investigation or surveillance of IPNs. There is no clear consensus or guidelines on the optimal staffing profile for nodule evaluation clinic or MDM, and this will vary depending on local infrastructure and resourcing. Reports describing LNET staffing profiles generally include, as a minimum, a specialist nurse, radiologist, and respiratory physician, with other reports including thoracic surgeons, medical and radiation oncology [14, 23, 33, 34, 35, 36]. The presence of a program coordinator within a LNET has been demonstrated to increase repeat CT compliance in the setting of screening [37]. The role of a specialist nurse is crucial and involves patient navigation, education, and support, coordination of care, advocacy, and shared decision making. Recommended LNET staffing profiles are presented in Table 1. Cross‐training of staff with experience in different specialities is encouraged.

## 4 Recommendations at Triage or Initial Assessment of Referral

This section is designed to support process and clinical decision making from initial triage through to clinic reviews and presentation at a nodule MDM.

**FIGURE 4:**
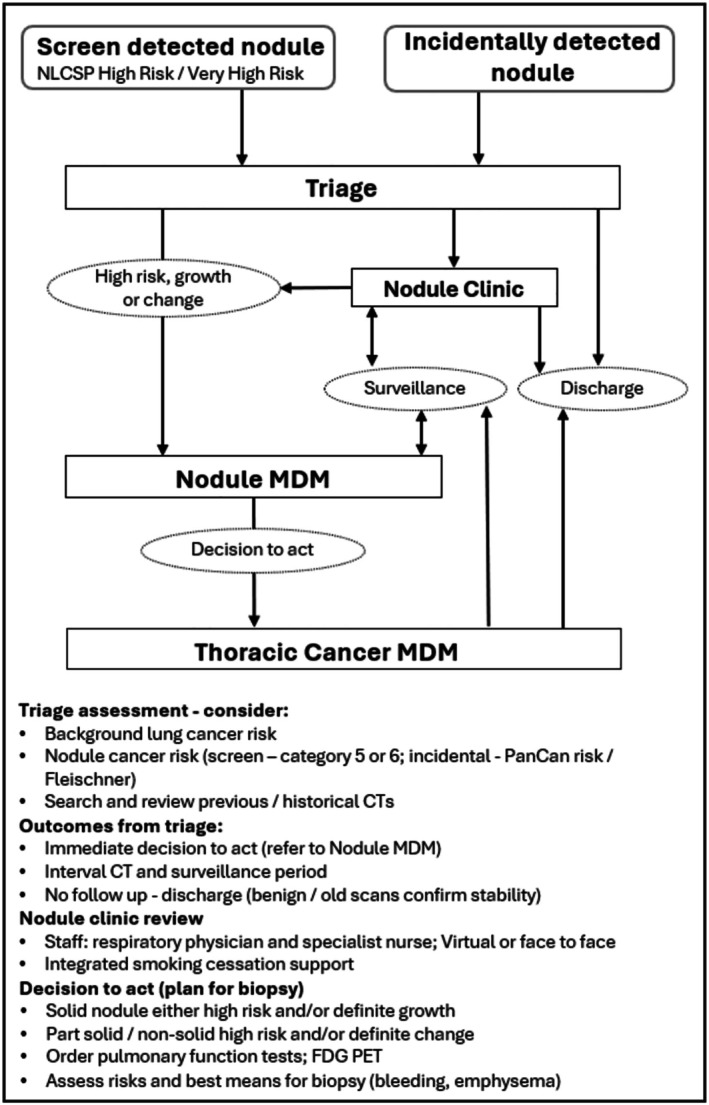
Considerations in the assessment of incidental and screen detected nodules. FDG‐PET, fluoro deoxyglucose positron emission tomography; NLCSP, National Lung Cancer Screening Programme.

Following receipt of a referral for an IPN, it is recommended that a respiratory specialist and specialist nurse experienced in nodule management oversee the initial assessment and management plan [18]. Multiple factors require consideration when evaluating an IPN, including assessment of an individual's background risk for lung cancer (very different between the incidental and screening populations), the nodule risk of cancer, and technical factors to be considered when interpreting CT images. Multidisciplinary decision making is required. Figure 4 provides a schematic overview of considerations in the assessment of an IPN.

**FIGURE 5:**
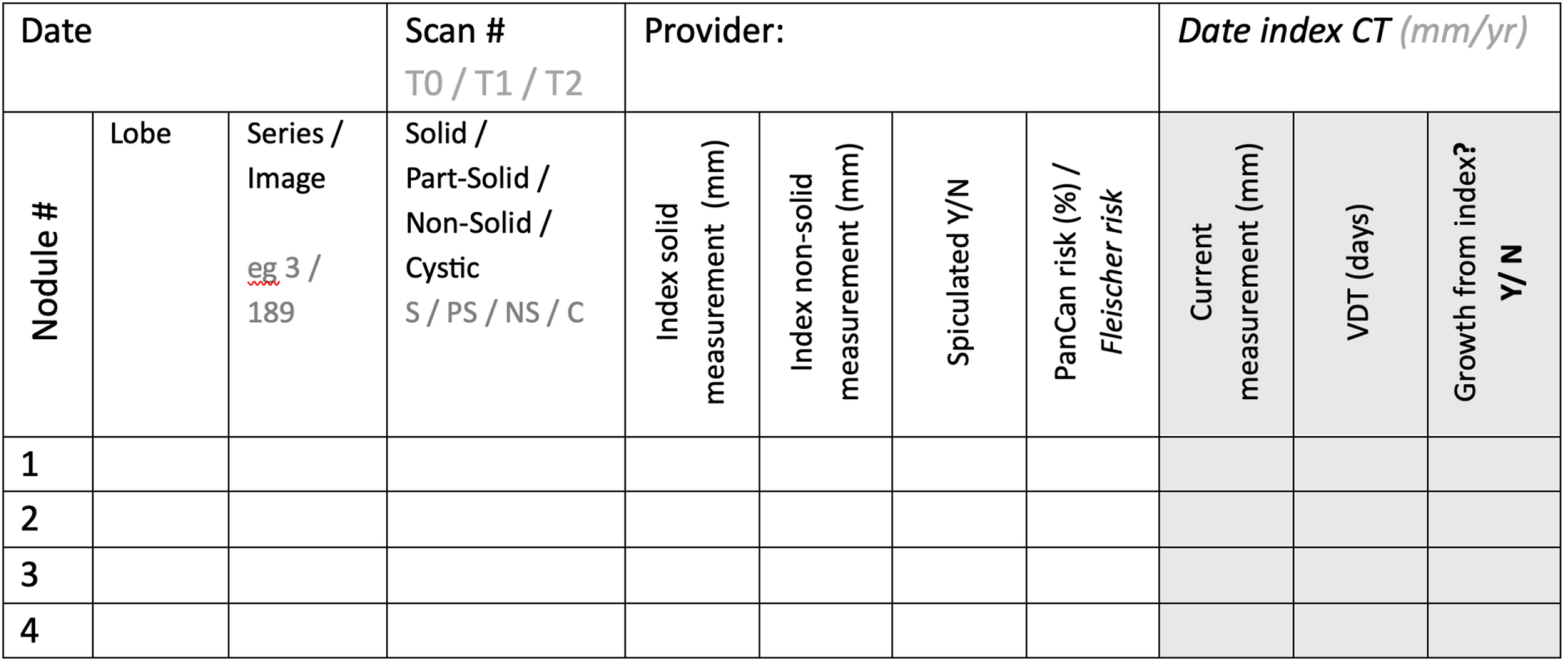
Suggested standardised supporting template for tracking indeterminate pulmonary nodules. VDT, volume doubling time. Spiculation is relevant for assessing PanCan risk; it forms only one component of the radiological likelihood of malignancy.

The use of standardised reporting templates has been shown to increase appropriate descriptions of IPNs [32, 38]. Therefore, a synoptic summary of nodules for follow‐up is recommended for the LNET to identify and track nodules over time, and act as a decision tool to prompt further investigation, as appropriate (see Figure 5 for an example). The use of image slice numbers together with which lobe (± segment) will help quickly identify nodules upon subsequent review. Liaison with reporting radiologists and use of key images are encouraged.

A clear record of the outcome of the review of every nodule is required to ensure comprehensive evaluation and to avoid missing a smaller growing nodule, as 20% of lung cancers in the Pan Canadian Early Detection of Lung Cancer study (PanCan) evolved from new or smaller nodules [39].

In the course of nodule assessment, additional (incidental) findings (such as emphysema or coronary artery calcification [40]) may arise that require additional investigation, referral(s), or management. These should be appropriately addressed in accordance with established clinical guidelines [41, 42]. The NLCSP Additional Findings Guidelines have been developed by the Australian and New Zealand Society of Thoracic Radiology (ANZSTR) for the Royal Australian and New Zealand College of Radiologists (RANZCR), for use in the screening (i.e., bi‐annual) setting [43]. These support standardised radiological recommendations for actionable additional findings.

## 5 Key Considerations When Reviewing Indeterminate Pulmonary Nodules

### 5.1 Comparison With Prior Imaging

Review of prior CT chest imaging is critical to assess the longitudinal behaviour of any IPN over time, as this critically informs malignancy risk. Hence, access to previous CT imaging is crucial. Any previously performed imaging should be sought for direct visual *side‐by‐side* comparison, rather than reliance on a report only. This may include thoracic imaging not targeting the lungs (e.g., CT thoracic aorta) or studies that partially image the lungs (CT cervical spine for lung apices, CT abdomen for lung bases) [3]. Previous chest x‐rays may be valuable (particularly for larger nodules), as well as other modalities, including magnetic resonance imaging or nuclear medicine studies performed with very low‐dose CT for anatomical localisation. Care should be taken when comparing between modalities, as technical differences will significantly affect nodule interpretation. Full characterisation of nodule composition is only possible on fine slice diagnostic CT.

When assessing nodule change over time, comparison should include all available prior studies and in particular the oldest available study (the ‘index CT’). Comparison only to the most recent CT is likely to underestimate growth or significant change in the lesion, particularly if nodule evolution has been slow or subtle.

Current best practice and guidelines recommend nodule surveillance periods as follows: solid nodules 2 years, part and non‐solid nodules for 5 years (see Table 2) [4, 24, 25]. Some cohort studies demonstrate growth of non‐solid nodules beyond 5 years and, on occasion, further longer follow up may be considered [44]. There should be deliberation of the balance of background risk of lung cancer, the patient's wishes and comorbidities.

### 5.2 Consideration of Technical Differences Between CT Scans

When comparing differing CT scans, it is important to account for technical differences, acknowledging limitations in reliably measuring slight interval growth due to inter‐scan and inter‐observer variability. Low‐dose CT is the most appropriate technique for nodule follow‐up, given radiation dose savings with preservation of clinically relevant nodule detail. Although the increase in image noise (graininess) related to this technique impacts the reliability of measurements, this is limited to nodules of low concern (< 5 mm) [45]. It is important to note differences in radiation dose (dose length product in mGy cm or CT dose index‐volume in mGy) when comparing studies.

Evaluation of CT imaging requires consideration of scan features that can influence nodule detection and characterisation. Key factors include CT scanner vendor/model, radiation dose, presence of contrast, phase of respiration, reconstruction method, slice thickness, and the software utilised for volumetric analysis.

When comparing nodules, nearby vascular branch‐points should be used to confirm the equivalence of a nodule between two studies. Acquisition in expiration will increase background lung density, which alters nodule appearance and may obscure nodules in atelectasis. This is also true for superimposed or adjacent transient pathology such as inflammation or consolidation.

Although the thinnest available CT slices should be examined, comparison of like‐thickness slices between studies is required. Nodules with a size approaching or less than the thickness of the image slice lose their crisp margin and may become faint or greyed‐out. Differences in slice thickness should be accounted for when assessing for nodule growth between studies.

### 5.3 Nodule Measurement, Size and Volume

The larger a nodule, the more strongly nodule morphology predicts malignancy over size measurements [46]. Nodules should be measured on the lung algorithm (sharp image presentation, with edge‐enhancement), using a lung window setting. Malignant features include internal pseudocavitation (bubbly lucencies), true spiculation (fine, closely spaced linear radiations), margin microlobulation, and distortion of surrounding architecture by the nodule. It is important to distinguish focal opacities likely caused by inflammation when considering the decision to act. Features of benign nodules are summarised in Table 2. Discussion with a radiologist will support appropriate nodule management.

The Fleischner Society guidelines recommend the average of long and short axis (bi‐dimensional) measurement in the largest plane [46]. Spiculations should not be included in the measurement. Error margins in diameter measurement may be up to 2 mm [47]. Measurement error is greater for irregular nodules of any density and for part‐solid nodules, where the solid and non‐solid components must both be measured [46].

Volumetry software measures nodule volume in cubic millimetres and is recommended when possible. It is not universally available and may not accurately segment nodules of complex morphology, such as part solid nodules or those abutting vessels. Reported error margins are up to 27% [48], with measurement variability seen in up to 11% of cases [49]. Because different software results in different intrinsic error, it is recommended to evaluate all studies for one patient using the same software [50]. Identical slice thicknesses and image reconstruction algorithms are recommended for greater accuracy when estimating volume changes between serial studies using volumetry software [51]. Volume assessment may be more reliable than two‐dimensional measurements in monitoring IPNs [47].

Volume doubling time (VDT) may be a useful metric for assessing pulmonary nodules, providing insights into growth rates that can indicate malignancy. Studies show that a VDT of less than 400 days is often associated with lung cancer, aiding in clinical decision‐making [52, 53]. A recent large systematic review and meta‐analysis of nearly 4000 patients with confirmed lung cancer demonstrated a pooled mean VDT for solid nodules of 207 days (95% CI 166–247), with slower rates for part solid and non‐solid nodules and for adenocarcinoma, compared to squamous cell and small cell lung cancer [52].

It is recommended that the LNET use VDT as part of nodule evaluation. To account for error margins, most guidelines define growth as more than 1.5 or 2.0 mm in diameter, or more than 25% in volume [46, 54]. Any nodule considered to be growing which is not clinically or radiologically suggestive of inflammation or infection requires discussion at a nodule MDM with decision to act for tissue biopsy as appropriate. Nodules up to 3 mm in size are recommended to be noted as ‘micronodules’ but not measured, given low cancer risk at this size and unreliability of measurement [46].

### 5.4 Use of Computed Assisted Detection/Artificial Intelligence in CT Interpretation

There is increasing evidence to indicate that accuracy in detection, measurement and characterisation of lung nodules is improved when radiologists are aided by computed assisted detection (CAD) and/or artificial intelligence (AI) software devices [55, 56].

Machine learning (AI) models have been available for several years with variability in regulatory approvals between jurisdictions. These AI tools commonly detect nodules, characterise them (in terms of size, density, spiculation), provide volume measurements, and offer serial volume comparisons between studies. The use of AI tools has been shown to reduce the rate of false negative interpretations by radiologists, although the tools may tend towards false positive results [57].

It is recommended for radiologists to use AI/CAD tools where possible when reporting for screening programmes to reduce the risk of missing nodules, improve the accuracy of measurements using volumetry, and provide additional information about VDT. The principles of clinically safe selection and deployment of AI tools are beyond the scope of this article and have been described elsewhere [58].

### 5.5 Assessing Malignancy Risk

Each individual nodule should be assessed for its risk of lung cancer. There are multiple guidelines for managing screen‐detected and incidentally detected IPNs; see Tables S1 and Figures S1 and S2 for a comprehensive summary and comparison.

Whilst most guidelines incorporate nodule size and risk in offering recommendations, there are differences in the applicable populations, size and risk determination, and thresholds for surveillance or investigation. Determining the most optimal approach requires appraisal of these key differences, clinical judgement, and clinician and patient preference. Box S1 summarises practical tips for clinicians in characterising nodule risk.

The Australian NLCSP will incorporate the PanCan risk model to determine nodule risk and inform interval scan recommendations [12]. As the PanCan model is derived and validated on baseline screening scans only, management of incident nodules (new from baseline) will be informed by nodule size and morphology, using a protocol that adapts features of Lung‐RADS to the Australian context [4]. It should be noted that decisions on interval imaging will be made by radiologists from within the NLCSP, not by hospital clinicians or primary care, with only high or very high risk nodules referred to respiratory teams linked to an MDM [12].

In the follow up of an incidentally‐detected IPN, for simplicity, this practical guide recommends the use of either the PanCan malignancy risk model [39] or Fleischner Society guidelines [3]. The PanCan risk tool was developed and validated in screening populations [39, 59] and has also been validated in incidentally detected nodules in retrospective studies [60, 61].

It is recommended that the LNET agree on a local protocol to ensure consistency; Table 2 lists recommended CT interval scan criteria, outcomes from initial review at triage, and characteristics of benign nodules that do not require assessment.

### 5.6 The Role of FDG‐PET for Nodule Evaluation

The test performance of fluorodeoxyglucose positron emission tomography (FDG‐PET) is highly dependent upon the pre‐test probability of malignancy in the individual and nodule, at least partially due to differences in nodule morphology, imaging and analysis techniques [62, 63, 64]. For solid nodules ≥ 8–10 mm, FDG‐PET may have utility in differentiating nodule pathology, disease staging and determining potential biopsy sites (with important caveats) [54, 65, 66].

A “negative” PET, or a nodule with a low standardised uptake value (SUV) max, should not be relied upon to inform malignancy risk for an IPN, particularly for nodules < 15 mm. Note that malignant non‐solid nodules and malignant part‐solid nodules with a small solid component would not be expected to exhibit a high maximum SUV.

Therefore, the utility of FDG‐PET for IPN management is usually *after* the decision to act to obtain a biopsy, as this decision should be influenced more strongly by the growth and morphology of the nodule, given the potential of falsely reassuring FDG‐PET findings in smaller solid nodules and part or non‐solid nodules.

## 6 Nodule Multidisciplinary Meeting

Any IPN assessed to be growing or having a high risk for malignancy should be discussed at a nodule MDM. The purpose of a nodule MDM is to improve guideline‐concordant care and decision making, ease the burden on thoracic cancer MDMs, and ensure consistent, safe, optimal, and timely care within any local infrastructure constraints. The minimum staffing profile should be that of the core LNET described above (respiratory physician, radiologist and specialist nurse), ideally with a care coordinator and specialty trainees.

### 6.1 Case Mix

Dependent on caseload and staff availability, a nodule MDM could discuss all cases referred to a service, or only nodules that are assessed to be growing, high risk, and/or where there is a perceived discrepancy with the radiology report and clinician's interpretation, see Table S2. A nodule MDM could be virtual, face to face, or hybrid, and more specialised centres could develop a local network to support smaller centres.

The nodule MDM should serve to support the decision to act by the clinician and should be the timepoint for the start of timeliness of care clinical quality indicators (CQIs) [67]. It also may be important and more efficient to distinguish a nodule MDM from the thoracic cancer MDM, which will have a different staffing profile, skill sets, and objectives [68]. Box 3 summarises the likely outcomes from a nodule MDM discussion.

BOX 3 **Outcomes from nodule MDM. MDM, multidisciplinary meeting; PET, positron emission tomography; PFT, pulmonary function tests.**

1

**Table:** 

**Outcomes from nodule MDM**
Any nodule considered to be growing, which is not clinically or radiologically suggestive of inflammation or infection, requires discussion at a nodule MDM with decision to act for tissue biopsy as appropriate.
Clinical impression—inflammatory/low risk/high risk/benign
Next steps—repeat CT (interval) / mode of biopsy/PET/PFTs/discharge
Recommendations needs clear documentation in medical notes and clear communication with patient and general practitioner
Documentation of clinical team present (medicolegal and financial rebate requirements)
Core data collection

The decision to act should be based around assessment of a nodule to be high risk and/or confirmation of definite growth or change (particularly for non‐solid nodules that may develop a solid component, but not change in size, or even reduce in size). See below for further considerations in the diagnostic assessment.

### 6.2 Recommended Core Data Collection for Nodule MDM

A nodule MDM presents an opportunity for clinical audit of outcomes and benchmarking against CQIs [17, 67]. It is recommended that outcomes such as malignancy rates, biopsy yield, complications, and time from decision to act to diagnosis are captured to facilitate clinical audit of practice. A full list of recommended variables is presented in Tables S3 and S4.

## 7 Workup of a Suspicious Pulmonary Nodule After a Decision to Act

This section aims to provide a practical guide and factors to consider in optimising the strategy for biopsy of an IPN after a decision to act has been made to obtain diagnostic tissue.

### 7.1 Diagnostic Considerations

Bronchoscopic and percutaneous techniques for biopsy are available, and the best approach is informed by patient factors, nodule characteristics, and local factors such as skill sets and available infrastructure. The nodule MDM provides the appropriate forum to discuss the optimal approach for obtaining tissue as discussed below.

### 7.2 Patient Factors Affecting Biopsy Decisions

Patient preference should be central to any medical decision‐making as this ensures that an individual's values are respected. Some patients may prioritise less invasive techniques or those with shorter recovery times [22, 69]. Techniques like CT‐guided lung biopsy carry a relatively higher (though small) risk of pneumothorax and thus a functional assessment and review of lung function (particularly FEV_1_) is strongly recommended. Risks are minimised by appropriate patient selection. Patients on anticoagulation therapy or with renal or hepatic disease are at increased risk of bleeding. Anticoagulation management should be carefully coordinated and documented with the patient's other clinicians (e.g., cardiologists in the patient with coronary stents) before and after the procedure. A meta‐analysis of > 2000 procedures demonstrated that when compared to endobronchial ultrasound (EBUS) bronchoscopy, CT guided biopsy is associated with a better diagnostic yield and accuracy when accessing small lesions and lesions that were proximal to the pleura, but a higher risk of complications [70].

Age, performance status, and overall health should also be considered where older patients or those with comorbidities may not be able to tolerate invasive procedures and their complications, and the least invasive technique may be preferable. Anaesthetic risk profile is also a consideration (general anaesthetic vs. conscious sedation vs. local anaesthetic alone).

On occasion, patients will present with IPNs that are too difficult or dangerous to biopsy or the patient is not fit for primary surgical excision–biopsy. In these cases, a full thoracic cancer MDM discussion for consideration of empiric ablative therapies, such as stereotactic radiotherapy, may be appropriate.

The choice of which procedure(s) to pursue should also consider the local availability and expertise within the institution. A workup checklist is presented in Table S5.

### 7.3 Nodule Characteristics Affecting Biopsy Decisions

There are multiple characteristics of an IPN that should be evaluated when considering a biopsy, including location, size, morphology, and molecular characterisation requirements from the tissue sample.

Peripheral nodules may be more accessible by percutaneous CT‐guided approach as compared with more central lesions [71]. A positive bronchus sign (a tubular area of hypoattenuation (airway) that leads directly into a peripheral pulmonary nodule) [72] increases radial EBUS (rEBUS) bronchoscopy yield from 44% to 55% without to 74%–81% [71, 73, 74]. For CT guided approaches, the risk of pneumothorax is increased when a CT guided needle crosses a fissure or emphysema [75].

Generally, larger nodules are easier to biopsy with most techniques. For instance, nodules > 2 cm biopsied using rEBUS have a higher diagnostic yield (75%–81%) than nodules < 2 cm (53%–64%) [73, 74, 76, 77]. Smaller nodules may therefore require advanced CT‐guided imaging or bronchoscopic navigation techniques for more accurate sampling. If multiple nodules are present, increased metabolic activity from FDG‐PET may guide which nodule to biopsy. Similarly, FDG‐PET may indicate that mediastinal sampling is required for concurrent tumour staging and an EBUS ± navigation bronchoscopy may be selected to facilitate both diagnosis and staging in a single procedure, rather than sequential CT guided biopsy followed by EBUS at a later time.

Non‐solid nodules may represent old scarring, atypical adenomatous hyperplasia or adenocarcinoma in situ, or early invasive malignancy [3, 78]. Therefore, factors that warrant consideration of nodule MDM discussion include sustained growth over sequential scans (> 1.5 mm in 12 months) [4], increase in nodule density or development of a solid component [3]. MDM decision making then requires careful consideration as to the likely underlying pathology and avoidance of overdiagnosis [79].

Finally, the requirement to obtain sufficient tissue for ever‐expanding molecular characterisation, including analysis for aberrations of *ALK*, *ROS‐1*, *PD‐L1*, *EGFR*, *KRAS*, *BRAF*, and *MET*, is increasingly crucial for all lung cancers. Techniques likely to yield the greatest tumour cellularity in a given situation are preferable, noting that it may be possible to run further tests on surgically resected specimens, if appropriate, see Table 1.

**TABLE 1:** Pathology tests to request on biopsy specimen order form.

Essential tests	Context specific tests
Cytology on needle aspirates, brushings and washings	Microscopy, culture sensitivity
Histopathology and immunohistochemistry stains, for example, TTF‐1, p40 on tissue biopsies such as forceps or cryobiopsies	Acid fast bacilli, MTB DNA probe, mycobacterial culture
For suspected NSCLC: – Molecular testing for routine targetable mutations. – Where possible, next generation sequencing for comprehensive tumour molecular analysis – Molecular studies can be requested on cytology and/or histology samples	Fungal culture, nocardia, cryptococcal stains
PD‐L1 immunohistochemistry staining—these can be requested on cytology and/or histology samples	

Abbreviations: DNA, deoxyribonucleic acid; MTB, mycobacteria tuberculosis; NSCLC, non‐small cell lung cancer; PD‐L1, programmed death ligand‐1.

### 7.4 Bronchoscopic Biopsy Techniques for Pulmonary Nodule Sampling

A comprehensive summary and overview of the potential advantages and disadvantages of different bronchoscopic techniques is presented in Table S6. In addition to the bronchoscopy biopsy technique, the biopsy tools employed, for example, forceps, transbronchial needle aspiration, cryoprobe, cytology brush can have an effect on demonstrating malignancy, the amount of tissue obtained and the need for further investigations. The availability of cytologists for Rapid On‐Site Cytological Examination (ROSE) has been shown to increase yield and reduce procedure time [80, 81, 82]. Linear endobronchial ultrasound (L‐EBUS) is a commonly used tool to sample mediastinal and hilar lymph nodes for diagnosing and staging lung cancer. L‐EBUS can, in some instances, sample peripheral nodules that lie adjacent to airways. Whilst this is not considered a primary pulmonary nodule biopsy tool, given the frequency of its use in lung cancer diagnosis and staging it is included in the summary.

Yield from currently available bronchoscopic navigation techniques is generally lower than for CT guided biopsies. rEBUS is the most widely used bronchoscopic navigation technique and yield based on a recent large meta‐analysis showed a pooled diagnostic sensitivity of 0.72 (95% CI 0.70–0.75) [83]. This may be increased slightly with the addition of a cryoprobe [84]. However, newer techniques, in particular cone‐beam CT guided navigation bronchoscopy, are available in several local centres and offer higher rates of diagnostic sensitivity for malignancy of 89.6 (95% CI 80–93) approaching that of CT guided biopsy [85].

Complication rates from bronchoscopic navigation techniques are generally low. Rates of pneumothorax from rEBUS based on meta‐analysis data involving 7601 patients were 0.7 (95% CI, 0.3–1.1) [83] and rates of bleeding in another meta‐analysis were 0.8% (61 bleeds in 7872 patients) [73]. Other, rarer complications which warrant consideration include post‐bronchoscopy pneumonia, airway/dental injury, cardiac and anaesthetic complications and require discussion during patient consent based on clinical circumstances.

### 7.5 Non‐Bronchoscopic Biopsy Techniques for Pulmonary Nodule Sampling

Percutaneous CT‐guided lung biopsy (CT fine needle aspirate (FNA) and core needle biopsy (CNB)) involves the use of real‐time CT imaging to guide a needle through the chest wall, across the pleura and into an IPN to obtain a biopsy. This technique is widely available in most centres with a very high diagnostic yield for peripheral nodules ~90% [86]. Overall, there is likely not much difference in yield and complications between FNA and CNB, although some studies show better material for molecular testing from CNB than with FNA [87]. ROSE is required to achieve optimal yields with CT‐FNA but not with CNB [87].

Potential complications of CT lung biopsy include pneumothorax requiring intercostal drain (5.6%) [75], haemoptysis (1.7%–4.1%) [75] and air embolism (rare, but serious) [88]. Factors that increase the risk of a CT‐guided lung biopsy pneumothorax include lesion location (> 2 cm depth, lower lobes), small lesion size, presence of emphysema, traversing pleura (e.g., multiple punctures crossing a fissure), longer procedure time, less proceduralist experience, and patient positioning with lesion side up [89]. Factors that increase the risk of CT‐guided haemorrhage include lesion location (> 4 cm depth), small lesion size, and pulmonary hypertension [87].

CT‐guided intervention is also used for the purpose of lung nodule marking, enabling surgical excision (typically wedge resection) of nodules that would be impalpable intra‐operatively [90]. This includes small or non‐solid nodules and can be performed using a metal microcoil (with or without intra‐operative fluoroscopy) or by hookwire placement.

Empiric surgical excision‐biopsy may be considered in settings where a biopsy is not possible due to technical factors, clinician and/or patient perceived risk is too high, or where MDM discussion considers the risk of malignancy sufficiently high that a negative biopsy would be considered a false negative. This approach provides a definitive diagnosis and simultaneous treatment; however, it is highly invasive, with longer recovery time, higher complication rates, and notably high risk of operating on benign nodules (benign nodule rate 13%–24% in published series) [91, 92]. Therefore, a full thoracic cancer MDM discussion is required for such cases.

### 7.6 Overdiagnosis

Overdiagnosis is defined as the detection, usually by screening, of a cancer that would not otherwise have become clinically apparent [93]. In this context, overdiagnosis occurs when either a benign IPN is treated as if it were cancerous, or if an indolent or slow‐growing IPN is treated in patients with advanced or significant comorbidities, with competing causes for death. Overdiagnosis leads to unnecessary procedures, patient anxiety, and harm. Screening studies estimate overdiagnosis rates of 18%–25% [94, 95], however, these were not implementation studies.

Steps to reduce overdiagnosis in the context of IPN evaluation may include: improved risk stratification—use of validated nodule risk prediction models can improve the likelihood of malignancy assessment; patient factors—thorough assessment of comorbidities, performance status, and patient preferences; surveillance—serial low‐dose CT scans can monitor growth or character change of low‐risk nodules (particularly part‐ and non‐solid nodules), avoiding immediate invasive procedures; multidisciplinary and LNET teams—collaboration among radiologists, respiratory specialists, and surgeons optimises management decisions; future innovations—use of companion diagnostics, such as circulating biomarkers (see below) may help mitigate overdiagnosis in future [96].

### 7.7 Future Directions

With the adoption of multiple LDCT screening programs globally, the understanding of, and literature on, management of IPNs is changing rapidly. In Australia, it is likely that the practical lessons from the implementation of the NLCSP will lead to changes in approaches to IPNs, especially given advances in diagnostic modalities, for example, robotic‐assisted bronchoscopy and transthoracic CT biopsy techniques.

Lung cancer screening in its current form will undoubtedly evolve as our understanding of lung cancer biology advances, particularly in population groups who are currently not captured by the current age and smoking eligibility criteria. There is a growing body of work into the role of biomarkers to inform an individual's risk of lung cancer [97]. Biomarkers have been predominantly explored in blood, but also from exhaled breath, saliva, sputum, bronchial lavage, and airway epithelium sampling. Potential biomarker candidates have included aberrations in the genome (circulating tumour DNA), epigenome (DNA methylation), transcriptome (circulating microRNA), and proteome (blood proteins, autoantibodies) [98]. The applications of such biomarkers may be to identify patients who may benefit from screening, regardless of age and smoking history. They may also facilitate risk‐stratification in individuals who have indeterminate pulmonary nodules detected by LDCT, thereby reducing the rates of false positive results and overdiagnosis [99]. However, much work is still required to ensure rigorous validation of potential biomarkers in screening populations.

### 7.8 Limitations

This project was not a formal systematic review or meta‐analysis and, therefore, formal graded recommendations are not provided. These pragmatic clinical guidelines are based on available evidence and best practice in an Australian context, and their incorporation into local practice should be guided by local experience and expertise, and available resources.

## 8 Conclusion

The timely, safe and effective management of both incidentally and screen detected pulmonary nodules is critical to realise the potential of early detection of lung cancer. A structured, multidisciplinary approach, including the establishment of lung nodule teams, clinics and MDMs, to streamline evaluation, reduce variability in care and improve patient outcomes is critical to reduce both under‐ and over‐investigation of IPNs. By adopting local protocols and evidence‐based guidelines, teams can ensure quality, efficient person‐centred care. The integration of specialist nurses enhances person‐centred care by providing navigation, proactive assessment and education, coordination and support at the time of need throughout the diagnostic and management process. Ongoing challenges, including variability in guideline application and resource constraints, underline the need for continuous audit and adaptation of local protocols. By fostering collaboration and innovation, healthcare systems can improve the process of early lung cancer detection for patients with indeterminate pulmonary nodules.

## Author Contributions

**Fraser Brims:** conceptualization (equal), data curation (equal), project administration (equal), writing – original draft (lead), writing – review and editing (lead). **Annette McWilliams:** data curation (equal), writing – original draft (equal). **Jonathan Williamson:** data curation (equal), writing – original draft (equal), writing – review and editing (equal). **Miranda Siemienowicz:** data curation (equal), writing – original draft (equal), writing – review and editing (equal). **Tracy L. Leong:** conceptualization (lead), project administration (lead), writing – original draft (equal), writing – review and editing (equal).

## Conflicts of Interest

F.B. and T.L. are co‐authors on this manuscript; they are series editors for this invited review series, and T.L. is an Editorial board member for Respirology. They were excluded from all editorial decision‐making related to the acceptance of this article for publication. The other authors declare no conflicts of interest.

## Supporting information


**Data S1.** Supporting Information.
