# Supplementary material for: The TSANZ Practical Guide for Clinicians in the Management of Screen‐ and Incidentally‐Detected Nodules
Source: Respirology. 2025 May 29;30(7):558–73. doi: 10.1111/resp.70065 (PMC12231763; doi:10.1111/resp.70065)
Supplement: Supplementary file 1 — Data S1. Supporting Information. [file RESP-30-558-s001.docx]

**The TSANZ practical guide for clinicians in the management of screen- and incidentally detected nodules**

**Online supplemental information**

**Indeterminate Pulmonary Nodule Guidelines Comparison**

**Solid nodules**

Figure 1 compares four major guidelines, including Fleischner society,^1^ British Thoracic Society,^2^ Lung-RADS^3^ and American College of Chest Physicians^4^ for the initial next investigation or surveillance interval recommendation for solitary solid indeterminate pulmonary nodules (IPNs). Management of multiple pulmonary nodules was excluded in this evaluation. NCCN offers two separate guidelines NCCN incidental nodule guidelines are based on the Fleischner society guidelines and the NCCN screened nodule guidelines are based on the Lung-RADS guidelines, although subtle differences are present.^4^ Due to significant overlap, the NCCN guidelines were not included in this comparison.


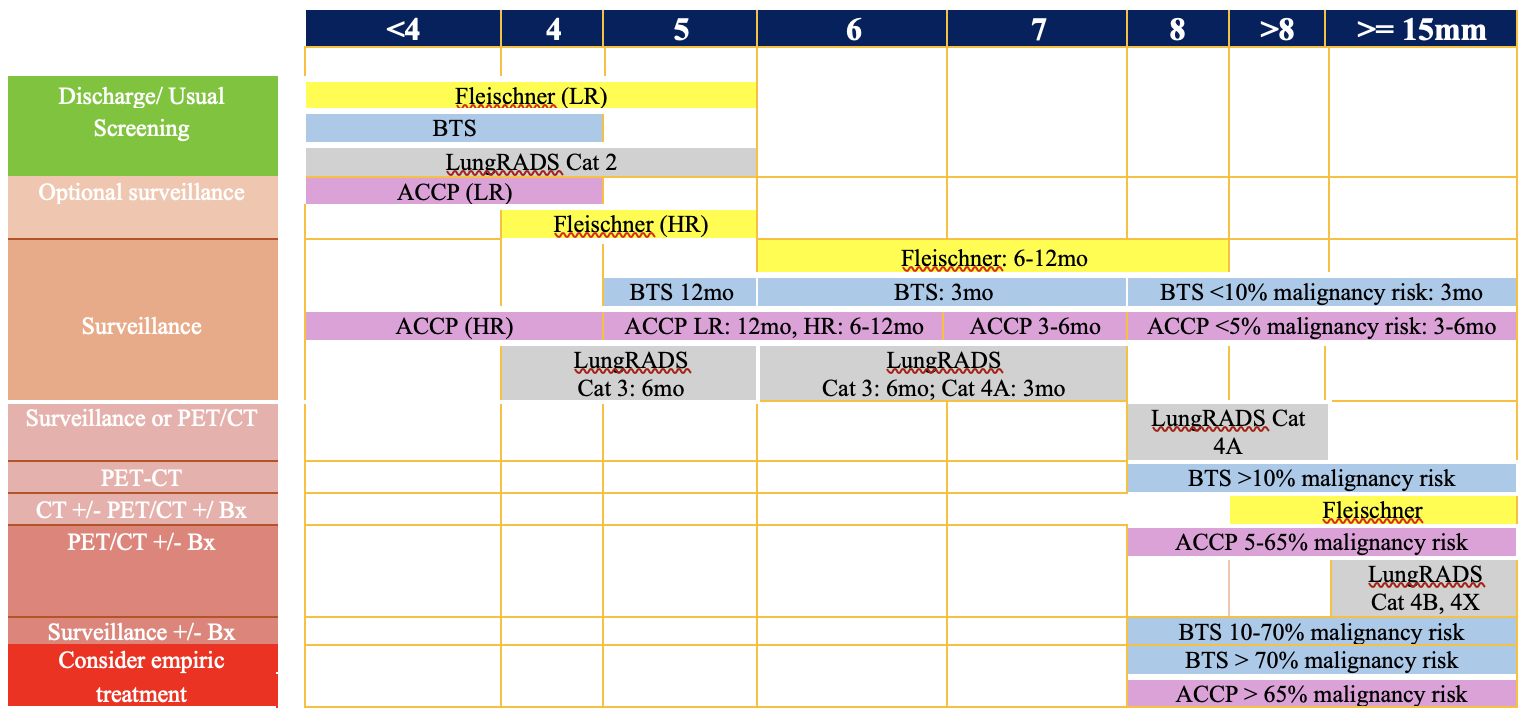


**Figure S1.** Comparison of management guidelines for solitary indeterminate solid pulmonary nodules. *BTS = British Thoracic Society; ACCP = American College of Chest Physicians;* *LR = low risk; HR = high risk; mo = months; Cat = category*; *PET = positron emission tomography;*

**Part solid and non-solid nodules**

Figure 2 compares four major guidelines, including Fleischner society,^1^ British Thoracic Society,^2^ Lung-RADS^3^ and American College of Chest Physicians^4^ for the initial next investigation or surveillance interval recommendation for solitary part solid and non-solid IPNs. Management of multiple pulmonary nodules was excluded in this evaluation. NCCN guidelines were excluded in this comparison again. Recommendations are depicted separately for pure ground glass nodules (GGN) and part-solid nodules (PSN) when the guidelines offered divergent recommendations.


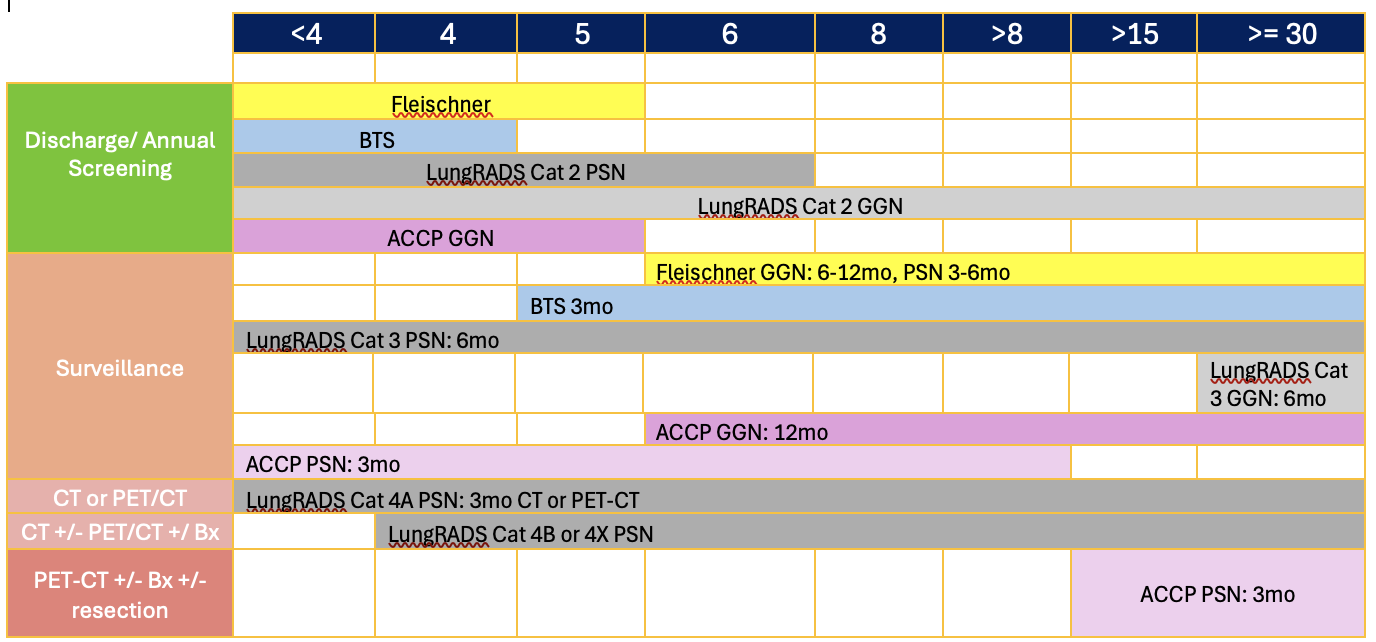
**Figure S2.** Comparison of management guidelines for solitary subsolid pulmonary nodules. *BTS = British Thoracic Society; ACCP = American College of Chest Physicians;* *GGN = ground glass (non-solid) nodule; PSN = part solid nodule; mo = months; Cat = category*; *PET = positron emission tomography;*

Table S1 compares four major guidelines, including Fleischner society, British Thoracic Society, Lung-RADS and American College of Chest Physicians. Comparison elements include applicable cohorts, recommendations for image acquisition, recommendations for nodule and growth measurement, risk assessment protocols and surveillance durations.

|  | Fleischner 2017^1^ | BTS 2015^2^ | ACCP 2013^4^ | Lung-RADS v2022^3^ |
| --- | --- | --- | --- | --- |
| Remit | Incidental | Incidental, Screened | Incidental, Screened | Screened |
| Guideline exclusions | Age < 35 years  Pre-existing malignancy  immunocompromise  screening population | Age < 18 years  Nodule has a pathological diagnosis of malignancy    *Known malignancy or immunosuppression not excluded | Not specified | Patients out of the screening cohort    *Lung-RADS assessment can still be considered for people no longer eligible for screening due to ageing or quit years beyond 15 years. |
| Imaging specifics | CT slices <= 1.5mm    Low risk nodules > 1cm visible on CXR can be followed up with CXR    Incomplete scans can be used for nodule assessment: if < 6mm (low risk), formal CT chest not required. If nodule 6-8mm, CT chest can be performed at next interval time. If > 8mm, formal CT chest is required. | CT slides <= 1.25mm | For nodules >8mm, PET-CT for nodule characterisation is preferred for nodule risk 5-65%, but not for risk > 65%. PET-CT can be performed for staging/ pre-treatment workup.    Surveillance is preferred when nodule risk is <30-40% and PET-CT does not show hypermetabolic activity or dynamic contrast CT does not enhance > 15 Hounsfield units. | CT chest with contrast is recommended for category 4A, 4X when further imaging/ investigation is warranted.    Specific recommendations for atypical cysts, juxtapleural nodules, airway-centred nodules and potentially infectious findings. |
| Size measurements | Average of long and short axis in plane with longest dimensions rounded to whole number | Volumetry preferred  Maximal diameter in any plane | As per Fleischner | Average diameter. Measurement and reporting to one decimal point. |
| Growth definition | >= 2mm in diameter | > 25% volume | Not specified | >1.5mm in mean diameter within a 12 month interval  ≤1.5mm growth in a >12 month interval is defined as slow-growing |
| Diameter to volume definitions | 6mm = 100mm^3^  8mm = 250mm^3^ | 5mm = 80mm^3^  8mm = 300mm^3^ | Not specified | 4mm = 34mm^3^  6mm = 113mm^3^  8mm = 268mm^3^ |
| Risk assessment | Cutoff for discharge selected as <1% malignancy risk    Low risk classified as <5% risk High risk ≥5%    ACCP nodule risk calculation recommended | PanCan risk calculator used (<10% low risk)    PET-CT and Herder model recommended if PanCan risk>= 10%.    Risk categories: low <10%, intermediate 10-70%, high >70% | Clinical judgement or a validated quantitative model for risk assessment recommended (e.g. Mayo).    Qualitative features on clinical history, CT, PET and biopsy provided in determining risk category.    Risk categories: low <5%, intermediate 5-65%, high >65% | Size-based risk  Risk modified by classification of nodule as pre-existing or new since last scan. |
| Solitary IPN surveillance duration | 2 years | 1 year if volume growth <25%  2 years if diameter used  Surveillance >2 years considered if VDT >400 days | 2 years | Annual screen |
| Non or part solid nodule surveillance | 5 years | 4 years | GGN: 3 years,  PSN: 3-5 years | Annual screen, unless >20mm |

**Table S1.** Summary of differences between various pulmonary nodule management guidelines. *British Thoracic Society; ACCP = American College of Chest Physicians; GGN = ground glass (non-solid) nodule; PSN = part solid nodule; PET = positron emission tomography;*

| **Different nodule MDM models of care** | **Potential advantages** | **Potential disadvantages** |
| --- | --- | --- |
| All nodules reviewed | - Enables consistency of practice - Does not rely on individual clinician - May be more useful for incidental nodules that have lacked the risk-based, protocol driven follow up of screen-detected nodules - All cases reviewed may ensure fewer cancers missed | - Time and staff resources - Inefficiency: low risk and stable nodules may be discussed |
| Only high risk / growing nodules reviewed after decision to act | - Allows focus on more complex cases - Efficiency – higher likelihood of intervention / biopsy plan - Fewer cases - less staff time and resources | - Relies on skilled, trained LNET staff at triage and clinics - Requires robust operating procedures/ protocol |

**Table S2**. Potential advantages and disadvantages of different nodule MDM models of care. *LNET = lung nodule evaluation team*

Tables S3 and S4 summarise the recommended core and desirable data required for a nodule MDM to capture the important clinical information required and ensure audit and benchmarking of clinical care.

| **Patient** | **Scan (Index)** | **Nodule** |
| --- | --- | --- |
| Name | Date | Lobe |
| Record number | Provider | Subsegment / Slice number |
| DOB | Type | Morphology (S / PS / NS / Cy) |
| Gender | **Scan (Current)** | Nodule measurement |
| Ethnicity | Date (interval from index) | Solid nodule measurement |
| PHx Lung Ca | Provider | Spiculation |
| FHx Lung Ca | Type | Radiological likelihood of malignancy |
| PHx any cancer | Number of nodules >4mm | Air bronchus sign |
| PHx any cancer last 5 years | Mediastinal lymphadenopathy | **Outcomes** |
| Immunosuppression | Emphysema on CT | Differential diagnoses |
| Smoking status | **Additional imaging** | **Recommendations** |
| Pack years | If PET: date / provider | Surveillance - Interval (months) |
| Asbestos / occupational carcinogen exposure | SUV max | Biopsy – modality:   - EBUS radial / linear / combined - CTFNA / CTCNB / U/S FNA / U/S CNB |
| TB exposure / history |  |  |
| ECOG performance status |  | Thoracic MDM discussion (consider empiric SABR / surgery) |
| Screen detected / incidental |  | Other test(s) |
| Clear question for the meeting |  |  |

**Table S3.** Core data for Nodule MDM. ‘Index scan’ refers to the first scan where the nodule was observed. *PHx = personal history; FHx = family history; TB = tuberculosis; ECOG = Eastern Cooperative Oncology Group; PET = positron emission tomography; SUV max = maximum standardised uptake value; S / PS / NS / Cy = Solid / Part-Solid / Non-Solid / Cystic; CT-FNA = CT fine needle aspirate; CT-CNB = CT core needle biopsy; U/S = ultrasound; EBUS = endobronchial ultrasound; SABR stereotactic ablative body radiotherapy; MDM = multidisciplinary meeting*

| **Lung Function** | **Nodule** |
| --- | --- |
| FEV_1_ | Distance from hilum/pleura |
| DLCO | Lobulation |
| **Scan (Index)** |  |
| Minimum thickness |  |
| **Scan (Current)** |  |
| Minimum thickness |  |

**Table S4**. Desirable data for Nodule MDM. *FEV_1_ = forced expiratory volume in 1 second; DLCO = diffusing capacity of the lungs for carbon monoxide*

| 1. **Assess reason for CT scan: screening vs. symptoms vs. incidental finding.**   - Screening – participant and nodule will have raised risk for malignancy - Incidental – assessment for background lung cancer risk required - Symptoms – consider short interval CT to assess for resolution |
| --- |
| 2. **Compare to old imaging. Find the earliest date the nodule was present (or absent)**   - Images should be compared with any previous imaging to establish a baseline and identify any significant changes in size (diameter or volume) over time. Evaluate any previous incomplete thoracic CT scans (e.g. CT coronary angiogram or CT abdomen that may have captured the area of interest). My Health Record may be useful (Australian setting) - Note differing CT scan characteristics known to affect nodule characterization. Key factors include, CT scanner vendor/model, radiation dose, presence of contrast, phase of respiration, reconstruction method, slice thickness and type of software utilised for volumetric analysis. - Changes in nodule size <1.5mm in diameter or <25% in volume are typically *not* considered to be demonstrating significant growth. |
| 3. **Assessment pre-test probability of malignancy for patient and the nodule**   - Patient factors include age, tobacco smoke exposure, family history, ethnicity, occupational exposures, history of other cancer. - The PanCan malignancy risk model incorporates clinical history and radiological parameters and is validated in screening and incidentally detected nodules. |
| 4. **Recognise limitations of PET-CT scans in nodule assessment.**   - Sensitivity and specificity of PET-CT for malignancy prediction is reduced for smaller solid nodules and subsolid nodules. Interval growth and nodule morphology may be a better predictor of malignancy in this context. |
| 5. **Refer to appropriate pulmonary nodule management guidelines for further investigation and surveillance intervals.**   - Agree a local protocol for incidentally detected nodules using the PanCan malignancy risk model or Fleischner guidelines. |
| 6. **Principles of care.**   - The optimal management of pulmonary nodules includes timely review, adherence to guidelines and multidisciplinary input for investigation and management (as part of a Lung Nodule Evaluation Team and Nodule MDM). - Streamlined referral pathways and Fast Track Clinics for urgent assessment of growing nodules can improve adherence to guidelines, timeliness of care and patient satisfaction. - A nodule assessment consult is an important opportunity to counsel on smoking cessation and manage other additional findings. |

**Box S1.** Practical tips to nodule characterisation and risk assessment. *PET = positron emission tomography*. *MDM = multidisciplinary meeting*

| **Process** | **Details** |
| --- | --- |
| Pre-procedure assessment | -Conduct a face-to-face or via telehealth consultation to assess patient’s medical history, medications, symptoms such as breathlessness and pain, and suitability for biopsy procedure and identify potential hazards.  -Provide procedure information and specific instructions eg. fasting time, anticoagulation advice.  -Review relevant imaging studies (CT, PET scan) for nodule localization and planning. |
| Consider investigations | -FBC, UEC, coagulation profile  -ECG, echocardiogram  -Lung function tests – prioritise prior to biopsy especially where a pneumothorax may not be well tolerated |
| Obtain informed consent and consider psychosocial factors | -Detailed discussion of the procedure, risks, pros and cons of the proposed technique(s), estimate of diagnostic yield and patient preparation.  -Address patient questions and concerns raised.  -Consider the psychosocial reaction to the decision to act and the possible distress associated with this and impact of waiting for results^5^ |

**Table S5:** Factors to consider in the workup for lung biopsy. *PET = positron emission tomography; FBC = full blood count; UEC = urea electrolytes & creatinine; ECG = electrocardiogram*

|  | **Description** | **Pros** | **Cons** | **Diagnostic yield**  **from Meta-analysis (95% CI)** |
| --- | --- | --- | --- | --- |
| **Flexible bronchoscopy** | Standard bronchoscopy performed with fluoroscopy in endoscopy/theatre complex | Minimally invasive. Low complication rate. Most useful for visible central nodules. Bronchus sign increases yield. | Reduced yield for peripheral and small nodules and for nodules not visualised on fluoroscopy. | Nodules <2cm 14-34%  Nodules >2cm 53-63%^6-8^ |
| **Ultrathin bronchoscopy** | Bronchoscope with a very thin insertion tube (approx. 3.1mm) and a working channel sufficiently to insert a rEBUS probe and biopsy tools such as FNA needles and 1.1mm cryoprobes | Minimally invasive. Low complication rate. Increased access to peripheral nodules. | Limited utility for central lesions | 56-82%  Meta-analysis: 65 (95% CI 60-70)%^9-12^ |
| **Radial Endobronchial Ultrasound (rEBUS)** | A 1.4mm diameter rotating ultrasound probe passed through the working channel of a standard or ultrafine bronchoscope into or adjacent to a pulmonary nodule to identify the lesion for biopsy | Minimally invasive. Low complication rate (pneumothorax <1%). Real time imaging. Suitable for small and peripheral nodules. Widely available. | Requires specialized equipment and training. Yield is lower for very peripheral nodules or nodules without a bronchus sign. | 51-92%  Meta-analysis: 72 (95% CI 70-75)%^13-15^ |
| **rEBUS with cryobiopsy** | A biopsy tool passed through the working channel of the bronchoscope to take frozen samples of the nodule | Minimally invasive. Larger tissue samples than forceps and FNA. Less crush artifact and better for molecular testing of samples. | Low rates of severe bleeding (<1%) and pneumothorax (2.4% requiring ICC) | 67-92%  Meta-analysis: 77 (95% CI 71-84)%^16-21^ |
| **rEBUS with electromagnetic navigation** | Uses electromagnetic guidance to navigate a bronchoscope through the airways to reach a peripheral nodule. Combines 3D imaging with real-time virtual guidance | Minimally invasive. Low complication rate. Real time imaging. Suitable for small and peripheral nodules. | High cost of equipment outlay and consumables per case. Limited availability. CT-body divergence* | 58-85%  Meta-analysis: 71 (95% CI 62-74)%^22-25^ |
| **rEBUS with virtual bronchoscopy** | Superimposes a virtual path onto the bronchoscopic image to guide navigation to the nodule. | Minimally invasive. Low complication rate. Suitable for small and peripheral nodules. | High cost of outlay. CT-body divergence* | 72-77%^26-29^ |
| **Linear Endobronchial Ultrasound (L-EBUS)** | A hybrid ultrasound and bronchoscope used to visualise structures beyond airway walls such as lymph nodes and masses. | Useful for lymph node sampling and central nodules. | Requires specialized equipment and training. Not suitable for peripheral nodules | For detection of lymph nodes: 37-96%^30, 31^ |
| **Augmented fluoroscopy (may also be used with rEBUS)** | A technique that overlays CT data and nodule position onto real-time fluoroscopic images during bronchoscopy to improve the localisation of otherwise fluoroscopically “invisible” or small nodules. | Minimally invasive. Low complication rate. Suitable for small and peripheral nodules. | Requires specialised software. Limited availability. CT-body divergence* | 73-82%^32-34^ |
| **Cone beam CT bronchoscopy** | Uses real time CT during bronchoscopy to identify the nodule then employs augmented fluoroscopy to identify the lesion for targeting. | High accuracy. Increased reach including for nodules without adjacent airways. Can target subcentimetre lesions. | Higher radiation exposure with longer screening times.  Learning curve for technique.  Longer procedure time. | 76-90%  Meta-analysis:78.5% (73.7-82.9)%^35-37^ |
| **Robotic assisted bronchoscopy** | Uses electromagnetic fields or “shape sensing” technology to navigate to and biopsy a nodule via disposable or limited use bronchoscopes with greater flexibility and reach than multiuse bronchoscopes. | Combines airway navigation, nodule “reach” technology, bronchoscope stability and ability to correct for CT-body divergence, into a single platform. When combined with integrated advanced imaging, RAB provides optimal diagnostic accuracy on par with CT guided transthoracic techniques with a lower complication profile. | Significant cost factors for initial outlay and ongoing consumables/service. Training requirements. | 79-94%  Meta-analysis: 87.6% (95% CI 81.3-89.5%)^38-41^ |

**Table S6.** Bronchoscopic biopsy techniques for pulmonary nodule sampling. note that head to head comparator studies between techniques are lacking, and direct comparison of yields between techniques based on the literature is therefore limited. *FNA = fine needle aspirate; rEBUS = radial endobronchial ultrasound;* *ICC = intercostal catheter; CI = confidence intervals; RAB = Robotic assisted bronchoscopy*

**CT-to-body divergence refers to the discrepancy between a nodule’s location on a pre-procedure CT scan and its position during real-time and is due to a variety of factors including the patient’s breathing phase and progressive atelectasis which can occur during procedures. The result is an apparent shift in the location of the nodule from where it appears on the CT scan.*

**REFERENCES**

1 MacMahon H, Naidich DP, Goo JM, Lee KS, Leung AN, Mayo JR*, et al.* Guidelines for management of incidental pulmonary nodules detected on CT images: from the Fleischner Society 2017. Radiology. 2017; **284**: 228-43.

2 Callister ME, Baldwin DR, Akram AR, Barnard S, Cane P, Draffan J*, et al.* British Thoracic Society guidelines for the investigation and management of pulmonary nodules. Thorax. 2015; **70 Suppl 2**: ii1-ii54.

3 Christensen J, Prosper AE, Wu CC, Chung J, Lee E, Elicker B*, et al.* ACR lung-RADS v2022: assessment categories and management recommendations. Journal of the American College of Radiology. 2024; **21**: 473-88.

4 Gould MK, Donington J, Lynch WR, Mazzone PJ, Midthun DE, Naidich DP*, et al.* Evaluation of individuals with pulmonary nodules: When is it lung cancer?: Diagnosis and management of lung cancer: American College of Chest Physicians evidence-based clinical practice guidelines. Chest. 2013; **143**: e93S-e120S.

5 Freiman MR, Clark JA, Slatore CG, Gould MK, Woloshin S, Schwartz LM*, et al.* Patients' Knowledge, Beliefs, and Distress Associated with Detection and Evaluation of Incidental Pulmonary Nodules for Cancer: Results from a Multicenter Survey. J Thorac Oncol. 2016; **11**: 700-8.

6 Rivera MP, Mehta AC, Wahidi MM. Establishing the diagnosis of lung cancer: Diagnosis and management of lung cancer, 3rd ed: American College of Chest Physicians evidence-based clinical practice guidelines. Chest. 2013; **143**: e142S-e65S.

7 Baaklini WA, Reinoso MA, Gorin AB, Sharafkaneh A, Manian P. Diagnostic yield of fiberoptic bronchoscopy in evaluating solitary pulmonary nodules. Chest. 2000; **117**: 1049-54.

8 Labbe C, Beaudoin S, Martel S, Delage A, Joubert P, Drapeau C*, et al.* Diagnostic yield of non-guided flexible bronchoscopy for peripheral pulmonary neoplasia. Thorac Cancer. 2015; **6**: 517-23.

9 Kim SH, Kim J, Pak K, Eom JS. Ultrathin Bronchoscopy for the Diagnosis of Peripheral Pulmonary Lesions: A Meta-Analysis. Respiration. 2023; **102**: 34-45.

10 Zheng X, Xie F, Li Y, Chen J, Jiang Y, Sun J. Ultrathin bronchoscope combined with virtual bronchoscopic navigation and endobronchial ultrasound for the diagnosis of peripheral pulmonary lesions with or without fluoroscopy: A randomized trial. Thorac Cancer. 2021; **12**: 1864-72.

11 Sehgal IS, Dhooria S, Bal A, Gupta N, Ram B, Aggarwal AN*, et al.* A retrospective study comparing the ultrathin versus conventional bronchoscope for performing radial endobronchial ultrasound in the evaluation of peripheral pulmonary lesions. Lung India. 2019; **36**: 102-7.

12 Oki M, Saka H, Asano F, Kitagawa C, Kogure Y, Tsuzuku A*, et al.* Use of an Ultrathin vs Thin Bronchoscope for Peripheral Pulmonary Lesions: A Randomized Trial. Chest. 2019; **156**: 954-64.

13 Steinfort DP, Vincent J, Heinze S, Antippa P, Irving LB. Comparative effectiveness of radial probe endobronchial ultrasound versus CT-guided needle biopsy for evaluation of peripheral pulmonary lesions: a randomized pragmatic trial. Respir Med. 2011; **105**: 1704-11.

14 Ali MS, Trick W, Mba BI, Mohananey D, Sethi J, Musani AI. Radial endobronchial ultrasound for the diagnosis of peripheral pulmonary lesions: A systematic review and meta-analysis. Respirology. 2017; **22**: 443-53.

15 Sainz Zuniga PV, Vakil E, Molina S, Bassett RL, Jr., Ost DE. Sensitivity of Radial Endobronchial Ultrasound-Guided Bronchoscopy for Lung Cancer in Patients With Peripheral Pulmonary Lesions: An Updated Meta-analysis. Chest. 2020; **157**: 994-1011.

16 Sryma PB, Mittal S, Madan NK, Tiwari P, Hadda V, Mohan A*, et al.* Efficacy of Radial Endobronchial Ultrasound (R-EBUS) guided transbronchial cryobiopsy for peripheral pulmonary lesions (PPL...s): A systematic review and meta-analysis. Pulmonology. 2023; **29**: 50-64.

17 Udagawa H, Kirita K, Naito T, Nomura S, Ishibashi M, Matsuzawa R*, et al.* Feasibility and utility of transbronchial cryobiopsy in precision medicine for lung cancer: Prospective single-arm study. Cancer Sci. 2020; **111**: 2488-98.

18 Brown M, Nguyen P, Jersmann H, Holmes M, Wong M. Radial Endobronchial Ultrasound-guided Transbronchial Cryobiopsy versus Forceps Biopsy for the Diagnosis of Solitary Pulmonary Nodules: A Prospective Randomised Trial. Open Respir Med J. 2023; **17**: e187430642309190.

19 Ankudavicius V, Miliauskas S, Poskiene L, Vajauskas D, Zemaitis M. Diagnostic Yield of Transbronchial Cryobiopsy Guided by Radial Endobronchial Ultrasound and Fluoroscopy in the Radiologically Suspected Lung Cancer: A Single Institution Prospective Study. Cancers (Basel). 2022; **14**.

20 Jiang L, Xu J, Liu C, Gao N, Zhao J, Han X*, et al.* Diagnosis of Peripheral Pulmonary Lesions with Transbronchial Lung Cryobiopsy by Guide Sheath and Radial Endobronchial Ultrasonography: A Prospective Control Study. Can Respir J. 2021; **2021**: 6947037.

21 Herath S, Wong C, Dawkins P, Veale A, Yap E, Williamson J*, et al.* Cryobiopsy with radial-endobronchial ultrasound (Cryo-Radial) has comparable diagnostic yield with higher safety in comparison to computed tomography-guided transthoracic biopsy for peripheral pulmonary lesions: An exploratory randomised study. Intern Med J. 2023; **53**: 1390-9.

22 McGuire AL, Myers R, Grant K, Lam S, Yee J. The Diagnostic Accuracy and Sensitivity for Malignancy of Radial-Endobronchial Ultrasound and Electromagnetic Navigation Bronchoscopy for Sampling of Peripheral Pulmonary Lesions: Systematic Review and Meta-analysis. J Bronchology Interv Pulmonol. 2020; **27**: 106-21.

23 Steinfort DP, Bonney A, See K, Irving LB. Sequential multimodality bronchoscopic investigation of peripheral pulmonary lesions. Eur Respir J. 2016; **47**: 607-14.

24 Lee B, Hwang HS, Jang SJ, Oh SY, Kim MY, Choi CM*, et al.* Optimal approach for diagnosing peripheral lung nodules by combining electromagnetic navigation bronchoscopy and radial probe endobronchial ultrasound. Thorac Cancer. 2024; **15**: 1638-45.

25 Pearlstein DP, Quinn CC, Burtis CC, Ahn KW, Katch AJ. Electromagnetic navigation bronchoscopy performed by thoracic surgeons: one center's early success. Ann Thorac Surg. 2012; **93**: 944-9; discussion 9-50.

26 Xu C, Wang Y, Li L, Yuan Q, Wang Y, Hu H*, et al.* Diagnostic Value of Virtual Bronchoscopic Navigation Combined With Endobronchial Ultrasound Guided Transbronchial Lung Biopsy for Peripheral Pulmonary Lesions. Technol Cancer Res Treat. 2021; **20**: 1533033821989992.

27 Bae S, Lim S, Ahn JJ, Jegal Y, Seo KW, Ra SW*, et al.* Diagnosing peripheral lung lesions using endobronchial ultrasonography with guide sheath: A prospective registry study to assess the effect of virtual bronchoscopic navigation using a computed tomography workstation. Medicine (Baltimore). 2020; **99**: e19870.

28 Chen ZB, Jin YP, Yu YM, Zhu DP, Ma HY, Chen L*, et al.* [A study of the diagnostic value of endobronchial ultrasound guide sheath transbronchial lung biopsy combined with virtual bronchoscopic navigation in peripheral pulmonary lesions]. Zhonghua Jie He He Hu Xi Za Zhi. 2016; **39**: 509-13.

29 Asano F, Ishida T, Shinagawa N, Sukoh N, Anzai M, Kanazawa K*, et al.* Virtual bronchoscopic navigation without X-ray fluoroscopy to diagnose peripheral pulmonary lesions: a randomized trial. BMC Pulm Med. 2017; **17**: 184.

30 Silvestri GA, Gonzalez AV, Jantz MA, Margolis ML, Gould MK, Tanoue LT*, et al.* Methods for staging non-small cell lung cancer: Diagnosis and management of lung cancer, 3rd ed: American College of Chest Physicians evidence-based clinical practice guidelines. Chest. 2013; **143**: e211S-e50S.

31 Ost DE, Ernst A, Lei X, Feller-Kopman D, Eapen GA, Kovitz KL*, et al.* Diagnostic yield of endobronchial ultrasound-guided transbronchial needle aspiration: results of the AQuIRE Bronchoscopy Registry. Chest. 2011; **140**: 1557-66.

32 Pritchett MA. Prospective Analysis of a Novel Endobronchial Augmented Fluoroscopic Navigation System for Diagnosis of Peripheral Pulmonary Lesions. J Bronchology Interv Pulmonol. 2021; **28**: 107-15.

33 Cicenia J, Bhadra K, Sethi S, Nader DA, Whitten P, Hogarth DK. Augmented Fluoroscopy: A New and Novel Navigation Platform for Peripheral Bronchoscopy. J Bronchology Interv Pulmonol. 2021; **28**: 116-23.

34 Pertzov B, Gershman E, Izhakian S, Heching M, Amor SM, Rosengarten D*, et al.* The LungVision navigational platform for peripheral lung nodule biopsy and the added value of cryobiopsy. Thorac Cancer. 2021; **12**: 2007-12.

35 Pritchett MA, Williams JC, Schirmer CC, Langereis S. Cone-beam CT-based Navigation With Augmented Fluoroscopy of the Airways for Image-guided Bronchoscopic Biopsy of Peripheral Pulmonary Nodules: A Prospective Clinical Study. J Bronchology Interv Pulmonol. 2024; **31**: 175-82.

36 Yu KL, Yang SM, Ko HJ, Tsai HY, Ko JC, Lin CK*, et al.* Efficacy and Safety of Cone-Beam Computed Tomography-Derived Augmented Fluoroscopy Combined with Endobronchial Ultrasound in Peripheral Pulmonary Lesions. Respiration. 2021; **100**: 538-46.

37 Yang H, Huang J, Zhang Y, Guo J, Xie S, Zheng Z*, et al.* The diagnostic performance and optimal strategy of cone beam CT-assisted bronchoscopy for peripheral pulmonary lesions: A systematic review and meta-analysis. Pulmonology. 2025; **31**: 2420562.

38 Fielding DI, Bashirzadeh F, Son JH, Todman M, Chin A, Tan L*, et al.* First human use of a new robotic-assisted fiber optic sensing navigation system for small peripheral pulmonary nodules. Respiration. 2019; **98**: 142-50.

39 Pyarali FF, Hakami-Majd N, Sabbahi W, Chaux G. Robotic-assisted navigation bronchoscopy: a meta-analysis of diagnostic yield and complications. Journal of Bronchology & Interventional Pulmonology. 2024; **31**: 70-81.

40 Pritchett M, Muller L, Ost D, Reisenauer J, Majid A, Simoff M*, et al.* Integration of shape-sensing robotic-assisted bronchoscopy and cone-beam CT for the biopsy of pulmonary nodules. Chest. 2021; **160**: A1622-A4.

41 Saghaie T, Williamson JP, Phillips M, Kafili D, Sundar S, Hogarth DK*, et al.* First‐in‐human use of a new robotic electromagnetic navigation bronchoscopic platform with integrated Tool‐in‐Lesion Tomosynthesis (TiLT) technology for peripheral pulmonary lesions: The FRONTIER study. Respirology. 2024; **29**: 969-75.
